# Editorial: Intestinal Homeostasis and Disease: A Complex Partnership Between Immune Cells, Non-Immune Cells, and the Microbiome

**DOI:** 10.3389/fimmu.2019.02775

**Published:** 2019-12-13

**Authors:** Marjorie De la Fuente, Thomas Thornton MacDonald, Marcela A. Hermoso

**Affiliations:** ^1^Innate Immunity Laboratory, Immunology Program, Faculty of Medicine, Biomedical Sciences Institute, Universidad de Chile, Santiago, Chile; ^2^Research Sub Direction, Academic Direction, Clínica las Condes, Santiago, Chile; ^3^Barts and The London School of Medicine and Dentistry, Blizard Institute, Queen Mary University of London, London, United Kingdom

**Keywords:** microbiome, gut microbiota, mucosal immunology, intestinal immunity, intestinal homeostasis, inflammatory bowel diseases, Crohn's diseases, ulcerative colitis

## Introduction

Epithelial cells, stromal cells, immune cells, and microbiota are fundamental components of the intestine, and all are importantin maintaining a healthy gut (1).

Risk factors that predispose an individual to loss of gut homeostasis include genetic variants and mutations, dysbiosis, immune dysregulation, and alterations in epithelial barrier function (2, 3). It is still unclear how these factors interrelate and lead to the development of inflammatory bowel diseases (IBD) in some individuals. Crohn's disease (CD) and ulcerative colitis (UC) are the most common types of IBD, affecting 1 in 250 individuals in the West. They are characterized by a relapsing and remitting progression of gut inflammation, which, if not treated with immune-suppression, leads to resection of inflamed tissue (4, 5).

The primary function of the gastrointestinal tract is to absorb nutrients and fluids to maintain health. Infectious agents take advantage of ingested food and water to either colonize the gut surface (e.g., *Vibrio cholera*) or invade the tissues (e.g., *Salmonella typhi*). The vulnerability of the gut is exacerbated by the fact that, in order to absorb nutrients, it is covered in a single layer of epithelial cells ~30 μm thick. The epithelial layer is protected by mucus from goblet cells, especially in the colon, and Paneth cells, which secrete anti-microbial peptides and lysozyme.

In this special issue of Frontiers in Immunology, we have collected original works and reviews that provide new insights into the role of intestinal homeostasis and disease, with an emphasis on immune and non-immune cells and the microbiome.

## A Focus on Epithelial Cells and Fibroblasts in the Gut

Curciarello et al. discuss the role of non-immune cells, such as epithelial cells and fibroblasts, in intestinal homeostasis and inflammation in the context of IBD. Epithelial cells are well-equipped to recognize infectious agents. They express Nod1 and Nod2 to recognize gram-positive and gram-negative infectious bacteria in the cytosol as well as TLRs on their apical and basolateral surfaces and in endosomes. Epithelial cells also express retinoic acid-inducible gene I to recognize RNA viruses. Additionally, they secrete cytokines that influence underlying cellular function, such as TGF-beta 1 and TSLP (6).

The fibroblasts that underlie the epithelium and that make up the stroma of the lamina propria maintain the structure of the lamina propria and make the matrix in which T cells, plasma cells, macrophages, and dendritic cells are embedded. In disease, however, when activated by pro-inflammatory cytokines, fibroblasts produce large amounts of interstitial collagenase and stromelysin, which degrade interstitial collagen and proteoglycans, respectively, leading to loss of epithelium and ulceration (7).

In the review by Wosen et al., emphasis is placed on how epithelial cells in the small intestine and lung mucosa constitutively express MHC class II. MHC II is not expressed in healthy colonic epithelium but is induced by pro-inflammatory cytokines in IBD. In diseases such as celiac disease, where there is very little TNF-alpha produced but a marked increase in interferon-gamma, MHC II increases on epithelial cells (it is well-known that interferon-gamma induces MHCII on non-professional antigen presenting cells). It is probable that antigen presentation mediated by MHC-II on epithelial cells is important in driving either tolerogenic or inflammatory responses.

Glal et al. show that activating transcription factor 3 (ATF3) is a key molecule needed to maintain intestinal integrity in health and inflammatory disease. ATF3-deficient mice die if given DSS-colitis, due to impaired epithelial regeneration. The authors suggest that IL-22 upregulates ATF3, leading to STAT3 phosphorylation by inhibiting phosphatase activity (SH-PTP2 and PTP-MEG2) and subsequent anti-microbial peptide production and epithelial fucosylation.

## A Focus on Innate Immune Cells in the Mucosa

Innate immune cells in intestinal mucosa are thought to be key modulators of tolerance and inflammatory responses. Stagg describes the heterogeneity of dendritic cells (DCs) in the gut and their role in regulatory and effector T cell-mediated responses in steady-state and inflammatory diseases. Bain and Schridde highlight the heterogeneity, ontogeny, origin, and inflammatory responses of intestinal macrophages. This is of great relevance given that, in contrast to other tissues such as the liver and brain where macrophages are yolk-sac derived and self-renewing, in the gut, for the most part, mucosal macrophages are bone marrow-derived.

Pantazi and Powell discuss the role of innate lymphoid cells (ILC) in the gut. They emphasize that Group 3 ILCs, which require RORγt for their development, are made up of diverse subpopulations. ILC3 has an important function in the response against pathogens, however dysregulated ILC3 activation may also promote inflammation, as implicated in IBD and/or colorectal cancer.

## Adaptive Immune Cell Responses in the Mucosa

Cells and molecules associated with distinct effector profiles are induced in the immune response characterizing IBD (8). The evidence is now quite overwhelming that CD is caused by an excessive Th1 response to gut microbes (9). Although IL-17A is also markedly over-expressed in CD, treatment of patients with an anti-IL-17A antibody exacerbated disease (10). For UC, the situation is still unclear. It was proposed that it was driven by an unusual type of NKT cell that made IL-13, but subsequent studies have not shown elevated IL-13 in UC, and two clinical trials with anti-IL-13 antibody in UC were both negative (11). Prostaglandin E2 (PGE2) production is known to play a role in intestinal inflammation and exert both pro-inflammatory and regulatory effects (12), but the mechanisms by which it affects T cell function during colitis are not clear. In this issue, Maseda et al. evaluate the effect of autocrine vs. paracrine (non-lymphoid) secretion of PGE2 in a murine model of T cell-dependent colitis. They demonstrate that lymphocytes contribute to PGE2 production and that microsomal prostaglandin E synthase-1 (mPGES-1, which drives PGE2 production) deficient CD4+ T cells were less able to induce colitis. In contrast, the authors suggest that mPGES-1 deficiency in non-lymphoid cells impairs FoxP3+ regulatory cell development in mesenteric lymph nodes and increases total CD4+ cell infiltration into colonic lamina propria. Together, these data provide evidence that T cell PGE2 signaling is required to restrain colitis.

Sorini et al. discuss the role of commensal bacteria-specific CD4+ T cells, highlighting the factors involved in the generation of these cells and their contribution to intestinal immune homeostasis and disease. Actually, the microbiota has been shown to promote pathogenic T cell expansion in IBD patients. In addition, the authors discuss in overview the most commonly used experimental model to study the induction of commensal bacteria-specific CD4+ T cells in mice, including gnotobiotic mice, soluble peptide-MHC tetramers, and TCR transgenic mice.

Coronado et al. present a novel model of diet-induced gut inflammation in zebrafish larvae, identifying changes in immune cell populations using a fluorescent reporter for macrophages, neutrophils, and T cells. They found that myelocytes (young granulocytes normally found in hematopoietic tissues) and T cells orchestrate gut inflammation in response to dietary proteins.

Tregs have been identified as a significant immunosuppressive population critically involved in maintaining intestinal homeostasis (13). Butera et al. studied the frequency and contribution of different Treg cells (CD3+CD4+Foxp3+ and CD3+CD4+LAP+Foxp3–) to the extent of disease in UC patients and a colitis mouse model. The authors concluded that CD3+CD4+LAP+ Tregs are responsible for limiting the extension of inflammatory lesions in UC.

Intraepithelial lymphocytes (IELs) play a key role in maintaining intestinal immune homeostasis (14). Zhang et al. describe original research on the role of Fas-associated protein with death domain (FADD) in IEL development. Dominant-negative FADD (FADD-DN) mutant mice have a selective deficiency of CD8αα+ TCRγδ+ cell IELs. Moreover, loss of γδ+ IELs in the gut of FADD-DN mice aggravates DSS-induced colitis, highlighting their important role in intestinal homeostasis.

Pararasa et al. studied memory B cells in blood and tissue samples of IBD patients and controls. The authors show a reduction in CD27-IgD- memory B cells in the blood of patients, while at the same time observing an increased frequency of this cell population in their mucosa.

## The Role of Microbiota and Dietary Components in Intestinal Homeostasis and Immune Response

The colon is colonized by more than 1 × 10^13^ bacterial cells, mostly of the phyla firmicutes and bacteroidetes (15). The microbiome shows major gradient differences along two axes, namely the mucosa-to-luminal axis and the longitudinal proximal intestine-to-distal intestine, with individuals showing substantial variations (15, 16).

Parada Venegas et al. discuss the production of short-chain fatty acids (SCFAs) by members of the commensal microbiota, their impact on epithelial and immune cells belonging to the innate and adaptive arms of the immune system, and their potential use as part of either pre- or probiotic therapies for attenuating gut inflammation.

Many factors have been shown to intervene with the gut microbiome, including age, genetics, diet, and drugs (15). The article by Siracusa et al. discusses the effect of the western diet (high intake of lipids, cholesterol, and salt) on the intestinal immune system, gut microbiota, and CD4+ T-associated cell differentiation into different effector sub-types. They suggest that a western diet increases the risk of intestinal and extra-intestinal inflammation and summarize the possible therapeutic effects of immune modulation using dietary supplementation with fiber, indoles, and vitamins.

Sugihara et al. summarize the role of different dietary nutrients in the maintenance or perturbation of immune intestinal homeostasis and highlight the relationship between nutrition and gut immune responses, including Th1/Th2/Th17/Treg cells, macrophages, and the microbiota.

## Evolutionary Biology and the Development of IBD and Cancer

Interdisciplinary research can greatly assist in understanding disease processes. Al Bakir et al. discuss the mechanisms underlying the connections between IBD and colon cancer risk from a mathematical/evolutionary perspective. They discuss the great opportunity to follow cancer evolution using genomic analyses over time of spatially separated colonic biopsies, from pre-malignancy to carcinoma, which show the key factors in the development of colorectal cancer (CRC). Clonal diversity and evolution could be useful for predicting progression risk, which will hopefully stimulate new thinking leading to novel early diagnosis and prevention strategies for IBD-associated CRC.

## Perspectives

Immune responses in lymph nodes and the spleen occur in a controlled plasma-rich environment where the levels of nutrients, hormones, and cytokines are strictly controlled. In the gut, by virtue of the special needs of the tissue, immune responses take place in an environment rich in exogenous immunomodulating molecules. The list is long, but in this issue, we have highlighted the role of diet, the microbiota, short-chain fatty acids, prostaglandins, aryl hydrocarbon receptor ligands, and vitamins. In this relatively brief series of articles, it was not possible to capture the full breadth of activity in mucosal immunology and inflammation in humans, but there is no doubt that this is one of the most exciting areas of immunology, with many surprises ahead.

## Author Contributions

All authors listed have made a substantial, direct and intellectual contribution to the work, and approved it for publication.

### Conflict of Interest

The authors declare that the research was conducted in the absence of any commercial or financial relationships that could be construed as a potential conflict of interest.
